# Epithelioid sarcoma of the pretragus: a rare pediatric entity with an unusual site

**DOI:** 10.1093/jscr/rjae606

**Published:** 2024-10-02

**Authors:** Rihane El Mohtarim, Samia Sassi, Nawal El Ansari, Naji Rguieg, Sara El Ghafouli, Lamiaa Rouas, Najat Lamalmi

**Affiliations:** Department of Pathology, Ibn Sina Teaching Hospital, Abderrahim Bouabid Avenue, Rabat 12000, Morocco; Mohamed V University, Nations unie Avenue, Rabat 12000, Morocco; Department of Pathology, Ibn Sina Teaching Hospital, Abderrahim Bouabid Avenue, Rabat 12000, Morocco; Mohamed V University, Nations unie Avenue, Rabat 12000, Morocco; Mohamed V University, Nations unie Avenue, Rabat 12000, Morocco; Pediatric Hematology-Oncology Service, Street Lamfadel Cherkaoui, Children’s Hospital, Rabat 12000, Morocco; Department of Pathology, Ibn Sina Teaching Hospital, Abderrahim Bouabid Avenue, Rabat 12000, Morocco; Mohamed V University, Nations unie Avenue, Rabat 12000, Morocco; Department of Pathology, Ibn Sina Teaching Hospital, Abderrahim Bouabid Avenue, Rabat 12000, Morocco; Mohamed V University, Nations unie Avenue, Rabat 12000, Morocco; Department of Pathology, Ibn Sina Teaching Hospital, Abderrahim Bouabid Avenue, Rabat 12000, Morocco; Mohamed V University, Nations unie Avenue, Rabat 12000, Morocco; Department of Pathology, Ibn Sina Teaching Hospital, Abderrahim Bouabid Avenue, Rabat 12000, Morocco; Mohamed V University, Nations unie Avenue, Rabat 12000, Morocco

**Keywords:** epithelioid sarcoma, head and neck, histopathology, immunohistochemistry, case report

## Abstract

Epithelioid sarcoma (ES) is a rare soft tissue tumor that is commonly misdiagnosed. It is a mesenchymal tumor that shows both mesenchymal and epithelial features. It tends to occur in the distal upper extremity in children and young adults but may appear in any location and any age group. Less than 1% of ES involve the head and neck. Clinically, the tumor can be mistakenly confused with a benign lesion as it can mimic nonspecific ulcers or infected warts. Histologically, ES is characterized by a nodular architecture and epithelioid appearance of cells centered with necrosis, mimicking a granulomatous process. We present the clinical history of a 12-year-old male who presented with an ES of the pretragus with a brief review of the literature to raise awareness on this rare entity and to discuss the challenges in managing histopathological differential diagnosis in front of this unusual clinical presentation.

## Introduction

Epithelioid sarcoma (ES) is a rare malignancy that was first described by Enzinger in 1970 [1]. It represents <1% of all adult soft tissue sarcomas and 4%–8% of pediatric non-rhabdomyoblastic sarcomas [2, 3]. ES usually presents as a painless, slow growing, and multinodular mass of the upper limb [4]. Morphologically, the classic type of ES mimics granulomatous processes with or without necrosis, mildly cellular atypia, and variable mitotic activity. In the pediatric population, the prognosis is usually good, although fewer nodal or distant metastases were described [5]. Herein, we describe the case of ES of the pretragus in a 12-year-old male with a brief review of the literature to raise awareness on this rare entity and to discuss the challenges in managing histopathological differential diagnosis.

## Case presentation

A previously healthy 12-year-old male presented with a slow growing, painless, and firm nodule of the left pre-tragus. The mass didn’t occlude the auditory tube, and the patient had not any hearing deficit or any symptom indicative of brain involvement.

**Figure 1:**
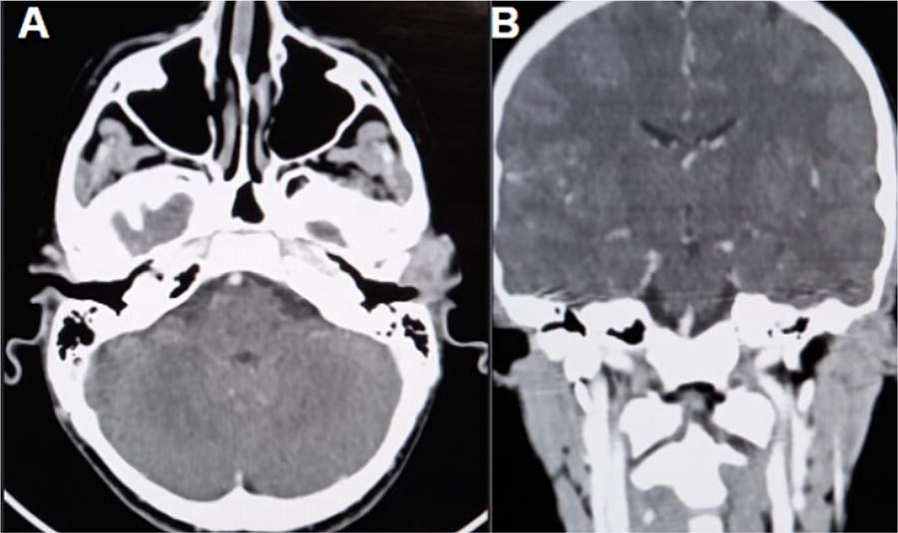
CT scan (A) axial section shows an ill-defined subcutaneous granulomatous process. (B) Coronal section of the process causing scalloping of the temporal bone.

**Figure 2:**
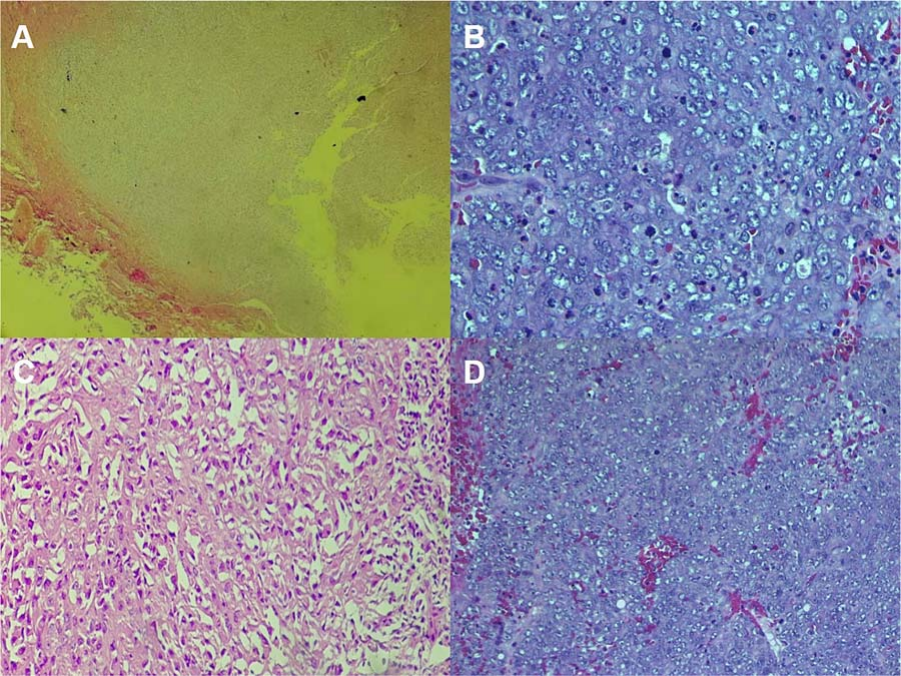
Histologic staining photomicrographs show multinodular architecture with central areas of necrosis (A; H-E stain; original magnification, ×40). Tumor cells are polygonal with eosinophilic cytoplasm, vesicular chromatin, and conspicuous nucleoli (B; H-E stain; original magnification, ×100). Areas of spindle-shaped cells (C; H-E stain; original magnification, ×200). Mitoses were noted (D; H-E stain; original magnification, ×400).

The CT scan showed an ill-defined, subcutaneous granuloma with heterogeneous enhancement, measuring 14.6 × 14.2 mm. The mass caused a smooth scalloping of the temporal bone without any locoregional spread or distant metastasis. Regional lymph nodes were free of tumors (Fig. 1). The patient underwent a complete surgical resection, and the mass was sent to our department for histological examination. Morphologically, the tumor showed a multinodular architecture with central areas of necrosis mimicking a granulomatous process. Tumor cells had a polygonal or spindled shape with eosinophilic cytoplasm, vesicular chromatin, and conspicuous nucleoli (Fig. 2). The stroma was myxoid, and some dystrophic calcifications were noted.

**Figure 3:**
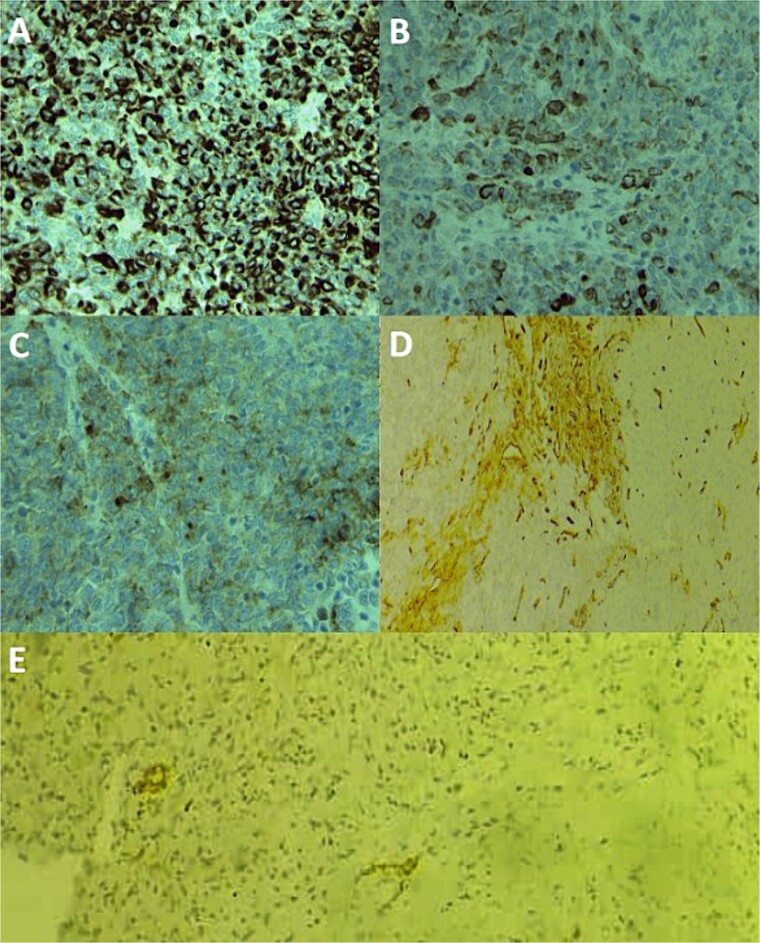
Immunohistochemical staining photomicrographs show cytoplasmic expression of cytokeratin AE1/AE3 (A; cytokeratin; original magnification, ×400), cytoplasmic expression of CK19 (B; CK19; original magnification, ×400), cytoplasmic expression of EMA (C; EMA; original magnification, ×400), focal membranous expression of CD34 (D; CD34; original magnification, ×200), and focal nuclear expression of ERG (E; ERG; original magnification, ×200).

A large panel was made on immunohistochemistry, showing positivity of tumor cells for pancytokeratin, CK19, EMA, CD34, ERG (Fig. 3), CD31 and SMA, loss of INI1 expression, and negativity for desmin, myogenin, S100, CD99, HMB45, and synaptophysin. Thus, the diagnosis of conventional ES was made, and other differential diagnoses were eliminated, including extrarenal rhabdoid sarcoma, melanoma, malignant peripheral nerve sheath tumors, and myoepithelial tumors. The patient was referred to the oncology department for radiotherapy.

## Discussion

ES is classified as a tumor with uncertain differentiation, and studies suggest a cocktail of epithelioid, histiocytic, fibroblastic, myofibroblastic, endothelial, and perineural differentiation [6]. Two principal types are described. Typically, classic ES predominantly targets the upper limbs of adolescents and young adults, between the ages of 10 and 40 years, with >60% arising in the fingers and hands [5]. The age of our patient was 12 years old, which was compatible with the data from the literature.

ES of the head and neck is very rare, and some cases were described in the ear and around it (supra-auricular, pre-auricular, and post-auricular locations) [7]. In our case, the mass was located in the pretragus region, making it an unusual site.

ES in the head and neck region can be mistakenly confused with carcinomatous processes, especially in young adults and older populations. Besides, what makes the diagnostic even more challenging is the expression of cytokeratin and epithelial membrane antigen (EMA) by ES [7].

It’s characterized by multinodular formations of epithelioid cells displaying minimal atypia and geographic necrosis. On the other hand, the proximal type is commonly deep and tends to affect the proximal limbs and limb girdles, as well as the midline of the trunk of middle-aged to older adults between 13 and 80 years [6]. This type exhibits sheets of larger, more pleomorphic cells. Both types present a male predominance, which is consistent with our case [6]. In 27% of cases, this tumor was associated with prior trauma at the site of the tumor. Despite their distinct presentations, both variants are aggressive tumors prone to local recurrence and eventual regional metastasis [6].

Clinically, classic ES usually manifests as painless, firm, slowly growing nodules on the hands and arms. These nodules often progress to chronic nonhealing ulcers with raised margins, which can mimic other ulcerative dermal processes. The proximal type presents as a deep soft tissue mass, commonly associated with hemorrhage and necrosis, and can reach considerable sizes up to 20 cm, with a median size of 4 cm [8].

There isn’t a specific CT scan or MRI feature for this tumor that could allow for an early, precise diagnosis. ES shows heterogeneous features encompassing fibrotic, granulomatous, necrotic, and cellular features, leading to variable T2-weighted signal intensities. T1-weighted images typically demonstrate isointensity compared to muscle. Suspicion should be raised in cases presenting multiple soft tissue nodules or persistent ulcers affecting the skin and subcutaneous tissues [9].

Microscopically, classic ES typically displays a distinct histological pattern characterized by nodular aggregates, sometimes appearing as nests or cords, composed of relatively uniform, plump epithelioid cells. These cells exhibit abundant eosinophilic cytoplasm and often feature prominent central zonal necrosis. In cutaneous lesions, ulceration may be observed. While cellular atypia is generally mild, recurrent or metastatic lesions may exhibit increased pleomorphism. Spindled cells may be present, typically merging with epithelioid cells without clear demarcation, although occasionally tumors may prominently exhibit a spindled morphology. Mitotic activity is usually present. In some cases, hemorrhage into spaces may occur, giving rise to an angiomatoid or angiosarcoma-like variant, while focal calcification or metaplastic ossification may also be present. Myxoid change is rare. Additional features include intracytoplasmic vacuoles, multinucleated giant cells, a storiform pattern, calcification, osseous metaplasia, chondroid metaplasia, lymphocytic reaction, vascular invasion, and neural invasion [5].

Microscopic findings in our case were compatible with a classic type ES rather than a proximal type, taking into consideration the presence of spindle cells, moderate to marked atypia, myxoid stroma, and some dystrophic calcifications. All those aspects are found in the classic type ES.

The proximal-variant ES is characterized by multinodular arrangements and sheets of large polygonal cells, featuring mildly to moderately pleomorphic vesicular nuclei with prominent nucleoli. Rhabdoid morphology is often observed, either focal or predominant. Central necrosis is frequent with or without geographic pattern as well as the ‘granulomatous’ appearance found in usual-type ES [5].

Immunohistochemically, ESs express variably low and high molecular cytokeratins, such as AE1AE3, Ck8, and CK19. They also show positivity for EMA, CD34, SMA, ERG, podoplanin, cyclin D1, FLI1, and HMB45. INI1 expression is lost in 90% of both types of ES [5].

To date, there is no specific cytogenetic abnormality detected in ES. However, frequent abnormalities of the chromosome 22q11, where SMARCB1 is located, suggest the implication of SMARCB1 loss in the tumorigenesis of ES [5].

The differential diagnosis is broad, including granulomatous processes, such as granuloma annular, rheumatoid nodules, or even infectious granulomatous reactions [10], carcinomas, melanoma, malignant peripheral nerve sheath tumor, extrarenal rhabdoid tumor, myoepithelial carcinoma, epithelioid angiosarcoma, and pseudomyogenic hemangioendothelioma [5].

Wide surgical excision is the first choice for treatment of ES [11]. Adjuvant or neoadjuvant radiotherapy is often indicated for localized tumors, to reduce local recurrence, which is frequent, especially with the proximal type [5].

Recurrence of the disease, lymph node metastases or distant metastases are more frequent in proximal type ES [5].

Proximal site, large size, advanced age, male sex, necrosis, vascular invasion, rhabdoid cytomorphology, and incomplete surgical excision are indicators for poor prognosis [5].

Generally, the prognosis in the pediatric population is better, with a 5-year overall survival of 92.4%.

## Conclusion

ES is a rare entity, and its occurrence in the pretragus is even more exceptional. The definitive diagnosis relies on histopathological examination, which remains a challenge due to the morphological and immunohistochemical similarities shared by ES, particularly with certain carcinomatous tumors and granulomatous processes.
